# Effective computational detection of piRNAs using n-gram models and support vector machine

**DOI:** 10.1186/s12859-017-1896-1

**Published:** 2017-12-28

**Authors:** Chun-Chi Chen, Xiaoning Qian, Byung-Jun Yoon

**Affiliations:** Department of Electrical and Computer Engineering, Texas A&M University, College Station, TX 77843 USA

**Keywords:** piwi-interacting RNA (piRNA), piRNA prediction, n-gram model (NGM), Support vector machine (SVM)

## Abstract

**Background:**

Piwi-interacting RNAs (piRNAs) are a new class of small non-coding RNAs that are known to be associated with RNA silencing. The piRNAs play an important role in protecting the genome from invasive transposons in the germline. Recent studies have shown that piRNAs are linked to the genome stability and a variety of human cancers. Due to their clinical importance, there is a pressing need for effective computational methods that can be used for computational identification of piRNAs. However, piRNAs lack conserved structural motifs and show relatively low sequence similarity across different species, which makes accurate computational prediction of piRNAs challenging.

**Results:**

In this paper, we propose a novel method, piRNAdetect, for reliable computational prediction of piRNAs in genome sequences. In the proposed method, we first classify piRNA sequences in the training dataset that share similar sequence motifs and extract effective predictive features through the use of n-gram models (NGMs). The extracted NGM-based features are then used to construct a support vector machine that can be used for accurate prediction of novel piRNAs.

**Conclusions:**

We demonstrate the effectiveness of the proposed piRNAdetect algorithm through extensive performance evaluation based on piRNAs in three different species – *H. sapiens*, *R. norvegicus*, and *M. musculus* – obtained from the piRBase and show that piRNAdetect outperforms the current state-of-the-art methods in terms of efficiency and accuracy.

## Background

The Piwi-interacting RNA (piRNA) is a new class of small non-coding RNAs (ncRNAs) whose functions are not fully understood. Recently, the studies have shown that piRNAs are associated with control of transposon silencing, transcriptional regulation, and mRNA deadenylation [1–3]. The piRNAs interact with Piwi proteins to form RNA-protein complexes involved in silencing of retrotransposons and other genetic elements. Furthermore, piRNAs are found to be emerging players in cancer genomes, and hence to have potential clinical utilities [4, 5]. Thus, there is a prompt demand for identifying the novel piRNAs through effective computational methods due to their clinical prospect. However, piRNA detection is not straightforward since piRNAs lack conserved structure motifs and sequence homology between different species [6, 7].

The piRNAs are the largest class of small ncRNAs with a wide variety of sequences in size about 26-31 nucleotide bases [8, 9]. There are two major classes of approaches developed for piRNA detection. The first class utilizes sequence-based features to identify piRNAs [10, 11]. Betel et al. [10] found piRNAs have the tendency to have the nucleobase Uridine at the 5’ cleavage sites and identified piRNAs by checking the Uridine positions and its 10 upstream and downstream bases. However, the prediction based on the Uridine positions is not accurate and the classification accuracy is 61-72% for Mouse piRNAs. The K-mer scheme [11] can have a superior performance by checking the frequencies of K-mer strings. All 1,364 K-mers from 1-mer strings to 5-mer strings are included to predict piRNAs. Since most piRNAs are derived from genomic piRNA clusters [12–14], the second class utilizes the information on clustering locus for piRNA detection. Among the approaches based on clustering locus of piRNAs, proTRAC [15] can identify piRNA clusters and piRNAs from a small RNA-seq dataset through a probabilistic analysis of mapped sequence reads. Furthermore, piClust [16] uses a density-based clustering method to identify piRNA clusters without assuming any parametric distribution model. Besides, the sequence-based approach can further incorporate distinctive features to detect piRNAs. For example, piRPred [17] integrates both the features of K-mer string and clustering locus based on multiple kernel fusion.

In this paper, we propose a novel sequence-based piRNA detection algorithm, called piRNAdetect, which can be used to detect novel piRNAs in genome sequences. First, we adopt the n-gram models (NGMs) based on the seed sequences to efficiently classify the recognized piRNAs into the homologous families. By integrating NGMs into the sequence classification, it enables flexible exploration of different sequence motifs and patterns in a dataset. Based on the classified families, we can further build the corresponding NGMs and utilize the support vector machine (SVM) to detect the potential piRNAs. The performance results based on the piRNAs from distinct species in the piRBase [18] database demonstrate the efficiency and the accuracy for piRNA detection using piRNAdetect.

## Methods

The main task of piRNA detection is to identify novel piRNAs in genome sequences. To achieve this, we first adopt the n-gram model (NGM) to classify a given database of recognized piRNAs into families with similar sequence motifs. The NGM is a class of probabilistic models, widely applied in bioinformatics research, including protein identification [19, 20], RNA structure modeling [21], and genome sequence analysis [22]. Based on homologous sequences, the NGM can estimate the similarity between sequences with the tolerance for the potential variations involved with insertions, deletions, and substitutions in the nucleotide or amino acid sequences [22]. The NGM is an (*n*−1)th-order Markov chain model and each nucleotide or amino acid base in a sequence only depends on what the preceding (*n*−1) bases are. Therefore, the homologous likelihood for a sub-sequence with length *L* in the sequence $\underline {b}$ can be efficiently estimated by the following Eq. (1):

1 $$  R(\underline{b},k)= \log{P(b_{k+1,k+n-1})}+ \sum\limits_{i=k+n}^{k+L} \log{P(b_{i}|b_{i-n+1,i-1})},  $$

where *k* is the offset of the sub-sequence in $\underline {b}$, and *b*_*i*_ represents the *i* ^*th*^ base of the sequence $\underline {b}$ while *b*_*i,j*_ represents the sub-sequence (*b*_*i*_,*b*_*i*+1_,⋯,*b*_*j*_) in $\underline {b}$. Moreover, the likelihood $R(\underline {b},k+1)$ can be efficiently updated from $R(\underline {b},k)$ when scanning the sequence $\underline {b}$ to search for the homology.

For the sake of piRNA detection, we can first classify the piRNA sequences into homologous families through NGMs based on the seed sequences in the dataset. Based on the classified families, we can then build the corresponding NGMs for detection and further extract the features through the NGMs for an SVM to detect piRNAs. Based on this idea, we propose a novel piRNA detection method called piRNAdetect. The procedure for piRNA detection using piRNAdetect is detailed in the following subsections.

### Clustering sequences that share common motifs

For a given dataset of sequences, we can classify the sequences with similar motifs into a homologous family through the NGM based on the seed sequence. Since there exists a subset of piRNAs derived from repeat regions [23, 24], some piRNAs have common motifs with repeat sub-sequences. Hence the sequence with the highest (n-1)-grams frequency is first taken as a seed to collect sequences with the similar sequence motifs. Based on the seed sequence, we can estimate the state probability *P*(*b*_*k*+1,*k*+*n*−1_) and the transition probability *P*(*b*_*i*_|*b*_*i*−*n*+1,*i*−1_) of the sequence $\underline {b}$ from the statistics, and a pseudo-count is added in the statistics to model potential mutations. Furthermore, the maximum $R(\underline {b},k)$ for all the sub-sequences with length *L*, which is set to the minimum sequence length within the dataset, is taken as the homologous sequence similarity $S(\underline {b})$. To normalize the bias of the sequence content in the sequence classification, the Z-score is adopted as the final similarity measure of the given sequence with respect to the corresponding NGM:

2 $$\begin{array}{@{}rcl@{}}  Z(\underline{b})=\frac{S(\underline{b})-\mu}{\sigma}, \end{array} $$

where $S(\underline {b})$ is the sequence similarity of the sequence $\underline {b}$, and the parameters *μ* and *σ* are the average and the standard deviation of the sequence similarity over the statistical ensemble for the dataset. Lastly, those similar sequences with the *Z*-score $Z(\underline {b})\ge Z_{th}$ are collected as a homologous family if the collected sequence number *N*≥*N*_*th*_, where the parameters *Z*_*th*_ and *N*_*th*_ are predefined threshold values. The classified family is then extracted from the dataset, and the process to classify sequences into the homologous family is repeated until all sequences in the dataset are checked to be the potential seeds.

### Predicting piRNAs using NGM-based features

For the purpose of piRNA detection, we first update the NGMs based on the classified sequences with the similar process as in the sequence classification. For each classified family, the state probability and the transition probability with pseudo-counts are estimated for the corresponding NGM. Since we utilize the Z-score of the sequence similarity $S(\underline {b})$ to normalize the bias of sequence length and family sequence content, the statistical average and the standard deviation of the sequence similarity are computed based on 18,000 randomly generated sequences obtained from Monte Carlo shuffling simulation [25]. Moreover, the lengths of the test sequences in the statistical evaluation are ranged from 21 to 36 nucleotides with a step size of 5, and the Z-score of the sequence similarity can be further estimated by SVM regression analysis based on the statistical averages and the standard deviations. The LIBSVM package [26] is employed for SVM regression based on the *ε*-support vector regression models using the radial basis function (RBF) kernel. With the Z-scores of the sequence similarities from the NGMs with respect to the classified families, piRNAdetect incorporates those features to detect piRNAs based on the SVM classifier.

In order to train the SVM classifier for piRNA detection, the sequences are drawn from the piRBase [18] and Rfam database 12.1 [27, 28] to construct the datasets with positive samples and negative samples for training and assessment. For each sequence in the positive samples, the sub-sequence with the same length is randomly drawn from the Rfam database and is shuffled to be considered as the negative control sample. Based on the dataset, we can train a *c*-support vector classification (c-SVC) model using the RBF kernel through the LIBSVM package [26] to detect potential piRNAs and compute the confidence probability for piRNA detection in a given genome sequence.

## Results and discussion

To test piRNAdetect, the piRNAs from the piRBase database with length from 26 to 36 are randomly taken to test the performance using 5-fold cross-validation (CV) approach. In the 5-fold CV, the test samples are randomly partitioned into 5 equal sized folds, and each fold is in turn retained as the test data for the validation while the remaining 4 folds are taken as the training data. The piRNA detection performance is evaluated in terms of the accuracy (ACC)=$\frac {(\text {TP}+\text {TN})}{(\text {TP}+\text {TN}+\text {FP}+\text {FN})}$, the true positive rate (TPR)=$\frac {\text {TP}}{\text {TP}+\text {FN}}$, and the false positive rate (FPR)=$\frac {\text {FP}}{\text {TN}+\text {FP}}$. TP denotes the number of correctly identified piRNAs, and TN denotes the number of correctly identified negative samples. FP denotes the number of negative samples incorrectly identified as piRNAs, and FN denotes the number of piRNAs that are missed in the detection.

In order to apply the n-gram model to piRNA detection, the size of *n* needs to be less or equal to the length of the target string. Besides, the larger size of *n* is suitable for the sequences with longer common motifs while the smaller size of *n* is proper for the sequences with intensive variations. Since piRNAs are divergent in both their structure and sequence, the tetragram is used to have superior performance in piRNA detection with reasonable computational complexity. In the following discussion, the parameters in the clustering sequences are first tested to better realize the NGM for piRNA detection and then the performance of piRNAdetect is compared with the K-mer scheme [11] as well as piRPred [17] based on the piRNAs from various species. To simulate piRPred, the locus information for the positive sample is referenced from piRBase database while random loci are assigned to the negative samples.

### Evaluating the effectiveness of NGMs for detecting piRNAs

The piRNAs from *H. sapiens* with a total number of 32,826 sequences in the piRBase database are first tested for the parameters in NGMs. In order to test the effect of the parameters *Z*_*th*_ and *N*_*th*_ in the NGMs for piRNA detection with the different size of the test datasets, one parameter is taken as a control variable and the other parameter is varied to check the corresponding accuracy of piRNA detection. Besides, the sizes of the test dataset used for 5-fold CV are ranged from 2000 to 32,000 with a step size 2000.

**Fig. 1:**
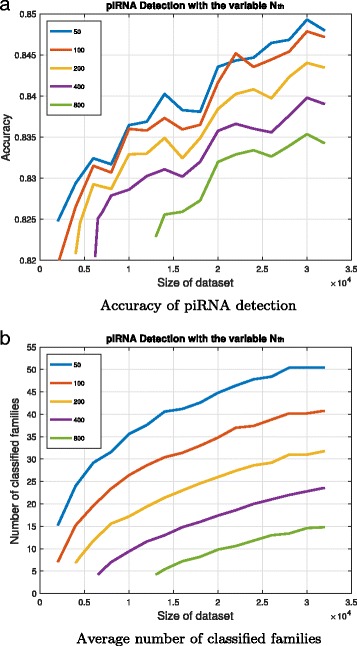
The piRNA detection accuracy and the average number of classified families for *Z*_*th*_=1.5. **a** The prediction accuracy is shown on the y-axis and the dataset size is shown on the x-axis. Lines in different colors correspond to different values of *N*_*th*_. **b** The average number of classified families for different *N*_*th*_ and dataset size

For the case with the fixed parameter *Z*_*th*_=1.5, Fig. 1 illustrates the accuracy and the average number of classified family with respect to the variable parameter *N*_*th*_ and the sizes of the dataset. The sequence classification needs the size of the dataset large enough to build the NGMs, and hence the classification with smaller *N*_*th*_ can build the NGMs easier and detect piRNAs in a smaller dataset. Moreover, when the size of the dataset increases, it can build more NGMs with the corresponding classified families and become more accurate in the detection since more motif patterns are recognized. In this case with piRNAs from *H. sapiens*, the piRNA detection with the parameter *N*_*th*_=50 has the highest possible accuracy. However, it also builds the maximum amount of the NGMs with the parameter *N*_*th*_=50 and the computational complexity is proportional to the amount of NGMs in both training and detection.

**Fig. 2:**
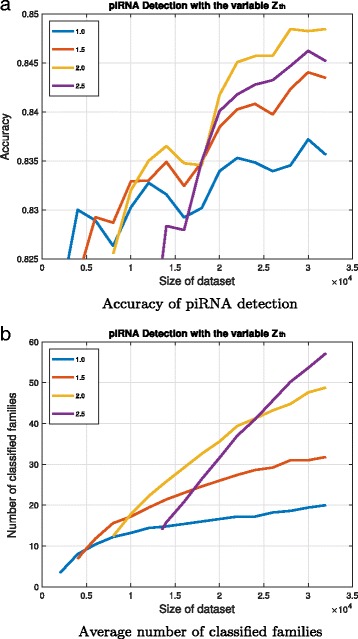
The piRNA detection accuracy and the average number of classified families for *N*_*th*_=200. **a** The prediction accuracy is shown on the y-axis and the dataset size is shown on the x-axis. Lines in different colors correspond to different values of *Z*_*th*_. **b** The average number of classified families for different *Z*_*th*_ and dataset size

For the case with fixed parameter *N*_*th*_=200, Fig. 2 illustrates the accuracy and the average number of the classified family with respect to the variable parameter *Z*_*th*_ and the sizes of datasets. The sequence classification with a higher threshold *Z*_*th*_ needs a larger dataset to build NGMs. With the size of the dataset large enough, the detection with a higher threshold *Z*_*th*_ can build more elaborate NGMs to characterize piRNAs and better improve the detection accuracy. However, the extremely high threshold *Z*_*th*_ can degrade the accuracy, and the piRNA detection with the parameter *Z*_*th*_=2.0 has the highest possible accuracy in this test case.

### Performance evaluation of piRNAdetect

To assess the piRNA detection performance of the proposed piRNAdetect algorithm, we perform 5-fold CV on the piRNAs from the species *H. sapiens*, *R. norvegicus*, and *M. musculus*. Moreover, the numbers of sequences for each species are listed in Table 1. We randomly drew 30,000 sequences from each species as the positive samples for the test datasets.

**Table 1:** Dataset size for each species

Species	Size
*H. sapiens*	32,826
*R. norvegicus*	63,182
*M. musculus*	51,664,769

In the following analysis, piRNAdetect utilizes the threshold parameters (*N*_*th*_,*Z*_*th*_)= (200, 1.5) to balance the performance and computational complexity. For performance comparison, the K-mer scheme [11] and piRPred [17] are also evaluated on the same test datasets. Table 2 summarizes the performance of piRNA detection by piRNAdetect, piRPred with default settings, and K-mer scheme with the cutoff parameter *t*=1.2 [11]. The accuracy of piRNAdetect for piRNA detection outperforms K-mer scheme and piRPred in all three distinct species. The piRPred algorithm uses loci information for piRNA detection and it may need a large dataset to make accurate predictions, as prediction schemes that utilize clustering locus typically require a large number of sequence reads to identify clusters.

**Table 2:** Prediction accuracy of piRNAdetect compared against the K-mer scheme and piRPred

Method	*H. sapiens*			*R. norvegicus*			*M. musculus*		
	TPR	FPR	ACC (%)	TPR	FPR	ACC (%)	TPR	FPR	ACC (%)
piRNAdetect	0.848	0.160	84.40	0.837	0.195	82.11	0.806	0.213	79.65
K-mer scheme	0.821	0.226	79.76	0.781	0.222	77.95	0.698	0.259	71.95
piRPred	0.375	0.098	63.85	0.290	0.201	54.42	0.208	0.020	59.39

**Fig. 3:**
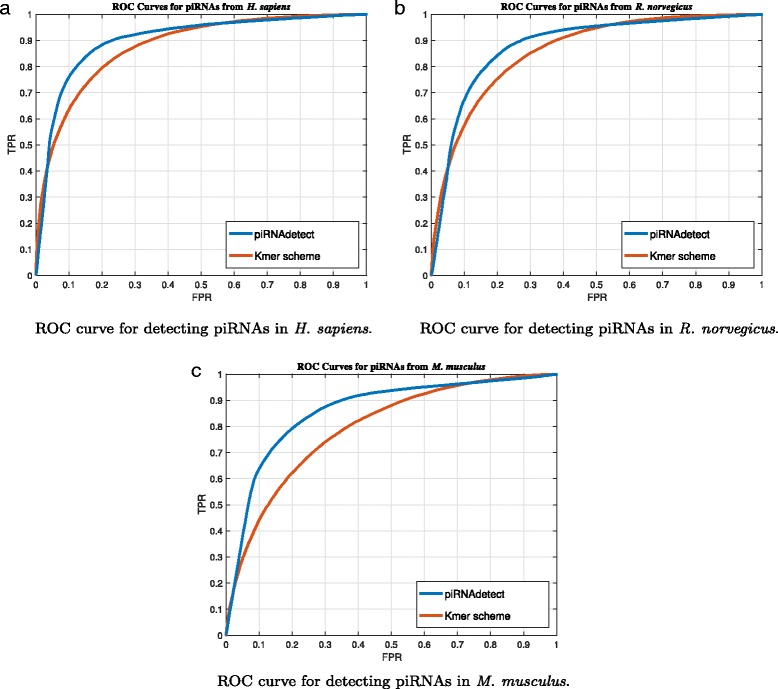
ROC curves showing the prediction performance of piRNAdetect and the performance of the K-mer scheme. **a** The performance for predicting piRNAs in *H. sapiens*. The false positive rate (FPR) is shown on the x-axis and the true positive rate (TPR) is shown on the y-axis. **b** The prediction performance for piRNAs in *R. norvegicus*. **c** The prediction performance for piRNAs in *M. musculus*

Since the cutoff parameter is introduced in the K-mer scheme to adjust the threshold in the decision, the receiver operating characteristic (ROC) curves for three species are also demonstrated in Fig. 3. Please note that the ROC curve for piRPred is not shown in the figure, as piRPred does not assign confidence probabilities to the predictions it makes. For comparisons based on ROC curves, the area under curve (AUC) can be used as a useful overall performance measure [29, 30], where a larger AUC indicates superior prediction performance. As summarized in Table 3, piRNAdetect clearly outperforms the K-mer scheme based on AUC.

**Table 3:** Prediction performance based on average AUC

	Average AUC		
species	*H. sapiens*	*R. norvegicus*	*M. musculus*
piRNAdetect	90.28	88.15	85.97
K-mer scheme	87.84	86.06	79.36

In general, the performance of piRNA detection depends on the characteristics of the training dataset and the prediction model that is constructed. For a sequence-based approach, the prediction method can achieve good performance if the sequences are regular and the dataset is large enough to be representative for all sequences. The K-mer scheme checks all possible sub-sequences with length *L*≤5 and extracts a total of 1364 features to detect piRNAs. In comparison, piRNAdetect can practically check longer sub-sequences while extracting a smaller number of useful features by utilizing NGMs. However, NGMs rely on the shared sequence motifs in the training dataset, hence their effectiveness will degrade if significant sequence motifs are absent or the dataset is not large enough to extract the representative sequence motifs. In this work, piRNAdetect extracts and utilizes less than 50 features based on NGMs for predicting piRNAs in *H. sapiens*, *R. norvegicus*, and *M. musculus*.

## Conclusions

The piRNAs lack conserved characteristics and prominent features that could be used for recognizing them, which makes accurate prediction of piRNAs challenging. In this paper, we proposed piRNAdetect, a novel algorithm for computational prediction of piRNAs. The proposed algorithm uses n-gram models (NGMs) to extract predictive sequence features for effective prediction of piRNAs. Besides, unlike piRPred, which is specifically designed for *Drosophila* and human data, our approach can be applied to identify sequences with shared sequence motifs for any given species. Comprehensive performance evaluation based on piRNAs in the piRBase database showed that piRNAdetect clearly outperforms the K-mer scheme, which is also a sequence-based scheme. Furthermore, despite the improved prediction accuracy, piRNAdetect utilizes a significantly smaller number of features compared to the K-mer scheme, which makes piRNAdetect more efficient and less prone to overtraining.
